# Anti-Osteoporosis Effects of the Fruit of Sea Buckthorn (*Hippophae rhamnoides*) through Promotion of Osteogenic Differentiation in Ovariectomized Mice

**DOI:** 10.3390/nu14173604

**Published:** 2022-08-31

**Authors:** Kun Hee Park, Joo-Hyun Hong, Seon-Hee Kim, Jin-Chul Kim, Ki Hyun Kim, Ki-Moon Park

**Affiliations:** 1 Department of Food Science and Biotechnology, Sungkyunkwan University, Suwon 16419, Korea; 2 School of Pharmacy, Sungkyunkwan University, Suwon 16419, Korea; 3 Sungkyun Biotech Co., Ltd., Suwon 16419, Korea; 4 Natural Product Informatics Research Center, KIST Gangneung Institute of Natural Products, Gangneung 25451, Korea

**Keywords:** *Hippophae rhamnoides*, C3H10T1/2, osteogenesis, anti-osteoporosis, ovariectomized mice

## Abstract

The fruit of *Hippophae rhamnoides* has been widely used for medicinal purposes because of its anti-inflammatory, antioxidant, antiplatelet, and antimicrobial effects. Since there are no clear reports on the therapeutic efficacy of *H. rhamnoides* in osteoporosis, this study aimed to confirm the potential use of *H. rhamnoides* for the treatment of osteoporosis through its osteogenic differentiation-promoting effect in ovariectomized mice. Through an in vitro study, we compared the effects of the EtOH extract of *H. rhamnoides* fruits (EHRF) on the differentiation of C3H10T1/2, a mouse mesenchymal stem cell line, into osteoblasts based on alkaline phosphatase (ALP) staining and the relative expression of osteogenesis-related mRNAs. The EHRF significantly stimulated the differentiation of mesenchymal stem cells into osteoblasts and showed 7.5 times (* *p* < 0.05) higher osteogenesis than in the untreated control. A solvent fractionation process of EHRF showed that the hexane-soluble fraction (HRH) showed 10.4 times (** *p* < 0.01) higher osteogenesis than in the untreated control. Among the subfractions derived from the active HRH by preparative HPLC fractionation, HRHF4 showed 7.5 times (* *p* < 0.05) higher osteogenesis than in the untreated naïve cells, and HRH and HRHF4 fractions showed 22.6 times (*** *p* < 0.001) stronger osteogenesis activity than in the negative control. Osteoporosis was induced by excision of both ovaries in 9-week-old female ICR mice for in vivo analysis, and two active fractions, HRH and HRHF4, were administered orally for three months. During the oral administration period, body weight was measured weekly, and bone mineral density (BMD) and body fat density were measured simultaneously using a DEXA machine once a month. In particular, during the in vivo study, the average BMD of the ovariectomized group decreased by 0.0009 g/cm^2^, whereas the average BMD of the HRH intake group increased by 0.0033 g/cm^2^ (* *p* < 0.05) and that of the HRHF4 intake group increased by 0.0059 g/cm^2^ (** *p* < 0.01). The HRH and HRHF4 intake groups significantly recovered the mRNA and protein expression of osteogenic genes, including ALP, Osteopontin, Runx2, and Osterix, in the osteoporosis mouse tibia. These findings suggest that the active fractions of *H. rhamnoides* fruit significantly promoted osteoblast differentiation in mesenchymal stem cells and increased osteogenic gene expression, resulting in an improvement in bone mineral density in the osteoporosis mouse model. Taken together, *H. rhamnoides* fruits are promising candidates for the prevention and treatment of osteoporosis.

## 1. Introduction

Osteoporosis is characterized by destruction of the bone microstructure and decreased bone mass, which increases the risk of fracture. According to U.S. statistics, approximately 30% of postmenopausal women suffer from osteoporotic fractures, which results in costs of approximately US$ 10 billion per year [1]. Osteoporosis is more common in women than in men and occurs mostly after menopause, when bone loss quickly increases [2,3]. The average age of menopause is 51 years, and it is generally caused by a reduction in female hormones due to ovarian malfunction [4,5]. Estrogen—a hormone secreted by the ovaries—plays an important role in the inhibition of bone resorption and has significant effects on bone formation. In particular, blood estrogen inhibits the destruction of bones through osteoclast differentiation, promotes the differentiation of osteoblasts that make bones, and protects bones by maintaining osteogenesis [6,7].

Osteoporosis medications are mainly divided into bone formation promoters and bone resorption inhibitors. Typically, bisphosphonates are used as bone resorption inhibitors, whereas parathyroid hormone is used as an osteogenesis stimulant [8]. Bisphosphonate is the most generally used therapeutic agent for the prevention and treatment of postmenopausal osteoporosis [9,10]. Selective estrogen receptor modulators are similar to estrogen, and selective estrogen receptor modulators (SERM) raloxifene increases bone mineral density, thereby reducing the risk of spinal fractures [11]. Hormone replacement therapy to supplement deficient female hormones caused by menopause is mostly used in combination with estrogen [12]. However, side effects of osteoporosis medications have also been reported. For example, bisphosphonates can cause side effects such as necrosis of the jawbone, atypical femoral fractures, and critical complications of bone necrosis [13,14]. In addition, selective estrogen receptor modulators increase the incidence of pulmonary embolism, high flush, and venous thromboembolism, whereas hormone replacement therapy has side effects including an increased risk of coronary artery disease, breast cancer, and stroke [15,16,17,18,19]. Therefore, treatment methods for osteoporosis must be developed using natural products with few side effects to overcome the above-mentioned problems.

*Hippophae rhamnoides* L. (Elaeagnaceae), commonly known as “Vitamin tree” and “Sea buckthorn”, is a spiny deciduous shrub. Historically, it has been widely used for medicinal purposes, particularly in southeastern and central Asia. In Chinese history, its pharmacological effect was identified more than a thousand years ago through records from the Tang and Qing dynasties [20]. Sea buckthorn oil was listed in the Russian pharmaceutical community in the 1950s and was used as a wound healing and anti-inflammatory agent [21]. Its fruits and bark have also been used in Tibetan folk medicine to treat cough, fever, pulmonary disorders, and tumors [22]. Several medicinal preparations based on *H. rhamnoides* have been used to treat oral inflammation, radiation damage, gastric ulcers, and burns; more than 300 preparations have been reported in the literature [23]. *Hippophae rhamnoides* bears yellow or orange berries, which are found as a powdered mix in food markets as a functional food and are used to manufacture fruit sauce and wine [24]. Vitamin C is the main nutritional component of *H. rhamnoides* fruits, the level of which surpasses that of lemons and oranges [25]. Previous studies on *H. rhamnoides* berries have reported that its extracts exhibit various therapeutic properties, including antiplatelet effects via the inhibitory mechanism of thrombin-activated platelets and antimicrobial effects through the inhibition of adhesion and biofilm formation of *S. aureus* and *C. albicans* [26,27]. Moreover, former phytochemical investigations on *H. rhamnoides* have proved the presence of varied types of bioactive substances, including amino acids, carotenoids, minerals, organic acids, phytosterols, polyunsaturated fatty acids, and vitamins [28,29]. Numerous researchers have attempted to determine the phytochemical components of *H. rhamnoides* fruits, identifying alkaloids, flavonoids, proanthocyanidins, and phenolic acids [30,31]. However, previous findings showed that only a few studies have been performed on the chemical components of *H. rhamnoides* fruits, despite their health benefits.

As part of an ongoing research project to discover the bioactivity potential of diverse natural sources [32,33,34,35,36], our group investigated the potential bioactivity of *H. rhamnoides* fruits. In our recent study of *H. rhamnoides* fruits, we identified a citric acid derivative, 1,5-dimethyl citrate, which showed anti-inflammatory effects by inhibiting the expression of pro-inflammatory mediators, iNOS and COX-2, and pro-inflammatory cytokines, IL-6 and TNF-α [37]. Our recent findings also provide experimental evidence that the organic acid derivatives present in *H. rhamnoides* fruits induce osteogenesis of mesenchymal stem cells (MSCs) and activate bone formation [38]. Although *H. rhamnoides* fruits have been shown to have promising compounds that induce osteogenesis by our group [38], no studies have shown that they effectively prevent bone loss caused by ovarian deficiency in ovariectomized (OVX) mice. Therefore, this study was conducted with the aim of confirming whether *H. rhamnoides* fruits effectively prevent bone loss in OVX mice and whether it can be considered as a possible therapeutic agent for the treatment of postmenopausal osteoporosis.

## 2. Materials and Methods

### 2.1. Plant Material

Freeze-dried *H. rhamnoides* fruits were purchased from Fuyang Bestop Import and Export, Ltd. (Fuyang City, Anhui, China). The material was authenticated by one of the authors (K.H.K.). A voucher specimen (HR-2021) was deposited in the herbarium of the School of Biotechnology, Sungkyunkwan University, Suwon, Korea.

### 2.2. Extraction and Isolation Fractionation

Dried *H. rhamnoides* fruit powder was extracted with 70% ethanol at 70 °C for 6 h. After filtering with filter paper, the extract was concentrated using a rotary evaporator and freeze-dried to obtain an EtOH extract of *H. rhamnoides* fruits (EHRF). The EHRF (1.1 kg) was suspended in distilled water (700 mL) and then sequentially followed by a solvent fractionation process three times with hexane, chloroform, ethyl acetate, and n-butanol. After concentration of the fractions, four main fractions with different polarities were obtained: hexane-soluble (20.8 g, HRH), chloroform-soluble (32.5 g, HRC), ethyl acetate-soluble (79.2 g, HRE), and n-butanol-soluble (256.8 g, HRB) fractions. The bioactive HRH fraction (20.8 g) was fractionated by preparative HPLC (MeOH/H_2_O, 80:20→100:0 for 60 min and 100% MeOH for 40 min) using an Agilent Eclipse C18 column (10 × 250 mm i.d., 5 μm, Agilent, Santa Clara, CA, USA) with a flow rate of 5.0 mL/min to obtain six subfractions (HRHF1-HRHF6) according on the separation of peaks. Preparative HPLC was performed on a Waters 1525 binary HPLC pump with a Waters 996 photodiode array detector (Waters Corporation, Milford, CT, USA). The six subfractions were concentrated using a rotary evaporator to obtain six subfractions: HRHF1 (1.2 g), HRHF2 (1.3 g), HRHF3 (1.1 g), HRHF4 (1.5 g), HRHF5 (1.1 g), and HRHF6 (1.3 g).

### 2.3. Cell Culture and Differentiation

The C3H10T1/2 cell line, derived from mouse embryonic fibroblasts, was cultured in Dulbecco’s modified Eagle’s medium with 10% heat-inactivated fetal bovine serum, 100 μg streptomycin, and 100 U penicillin at 37 °C in an incubator with 5% CO_2_. To measure osteoblastic differentiation, C3H10T1/2 cells were seeded at a density of 5 × 10^5^ cells/well in 6-well plates and treated with 10 mM β-glycerophosphate and 50 μg/mL ascorbic acid for 7–9 days. Oryzativol A (5 μM, 3.64 μg/mL), which has been proven to be effective in osteogenesis, was used as a positive control for osteogenic differentiation of the C3H10T1/2 cells [39].

### 2.4. Alkaline Phosphatase (ALP) Staining

The differentiated cells were fixed with 4% formaldehyde (Sigma-Aldrich, St Louis, MO, USA) for 30 min. After washing with 2 mM MgCl_2_ solution, cells were incubated with alkaline phosphatase (AP) buffer (100 mM NaCl, 100 mM Tris-HCl, pH 9.5, 10 mM MgCl_2_, and 0.05% Tween-20) for 15 min. They were then incubated in AP buffer containing 0.4 mg/mL nitro blue tetrazolium (Sigma-Aldrich, St Louis, MO, USA) and 0.2 g/mL of 5-bromo-4-chloro-3-indolyl phosphate (Sigma-Aldrich, St Louis, MO, USA). The reaction was terminated with 5 mM EDTA solution (pH 8.0). The degree of staining was measured at 405 nm using a SpectraMax M2 microplate spectrophotometer.

### 2.5. mRNA Isolation and Real-Time Polymerase Chain Reaction

Ribonucleic acid was isolated from the differentiated cells using the NucleoZOL reagent (Macherey-Nagel GmbH & Co KG, Duren, Germany). Complementary deoxyribonucleic acid (cDNA) was synthesized from 0.5 μg of total RNA using a ReverTraAce qPCR Master Mix kit (Toyobo, Osaka, Japan). The synthesized cDNA was mixed with an amplification mixture containing the THUNDERBIRD SYBR qPCR Mix (Toyobo, Osaka, Japan). cDNA was subjected to 40 amplification cycles of polymerase chain reaction (PCR) using a Thermal Cycler Dice (Takara, Kusatsu, Shiga, Japan) and normalized to 36B4 expression. The primer sequences were as follows: *Runx2*, 5′-CCC AGC CAC CTT TAC CTA CA-3′ (forward) and 3′-TAT GGA GTG CTG CTG GTC TG-5′ (reverse); *ALP*, 5′- GCA ACT TCC AGA CCA TTG GC-3′ (forward) and 3′-TCC CAC TGA CTT CCC TGC TT-5′ (reverse); *Osterix*, 5′- ATC TTC CAC TTC GCC TGC-3′ (forward) and 3′-AAC CAA TGG GTC CAG CAC-5′ (reverse); and *Osteopontin*, 5′-TGA TGA TGA CGA TGG AGA CC-3′ (forward) and 3′-GGG ACG ATT GGA GTG AAA GT-5′ (reverse). The above-mentioned process was also performed with the samples obtained by pulverizing both tibias of each mouse after animal sacrifice.

### 2.6. Western Blot Analysis

The differentiated cells were washed with cold phosphate buffered saline (PBS) and solubilized in lysis buffer (150 mM NaCl, 20 mM Tris-HCl (pH 7.5), 1 mM ethylenediamine tetra-acetic acid (EDTA), 1 mM ethylene glycol tetra-acetic acid (EGTA), 50 mM NaF, 2.5 mM sodium pyrophosphate, 1 mM b-glycerophosphate, 1 mM Na_3_VO_4_, 1% Triton X-100, and 1 mg/mL leupeptin). The samples were separated by sodium dodecyl sulfate-polyacrylamide gel electrophoresis and electroblotted onto a PVDF membrane. The electroblotted membrane was soaked in blocking solution (5% skim milk in Tris-buffered saline with 0.1% Tween-20) for 1 h at room temperature. Membranes were then incubated with each primary antibody overnight at 4 °C. Then, they were washed and incubated with the secondary antibody for 1 h. The blots were detected using an ibright 1500 (Thermo Fisher Scientific, Waltham, MI, USA). The relative level of osteogenic proteins was calculated with comparison to the level of *β-actin*, a housekeeping protein. The above-mentioned process was also performed with the samples obtained by pulverizing both tibias of each mouse after animal sacrifice.

### 2.7. Animals and Study Design

Healthy 9-week-old female ICR mice (Orient Bio, Korea) underwent either a sham operation (*n* = 6) or OVX (*n* = 30) under isoflurane anesthesia. Mice were housed in an animal facility under controlled temperature (22–24 °C) and humidity (50–60%) conditions with a 12-h light/dark cycle and free access to food and water. Both ovaries were excised in OVX-operated mice, and adipose tissue around both ovaries was excised from sham-operated mice. Mice were assigned to the following four groups: sham (*n* = 6), OVX (OVX-operation with vehicle administration; *n* = 10), HRH (OVX-operation with hexane-soluble fraction from the EtOH extract of *H. rhamnoides* fruits; *n* = 10), and HRHF4 (OVX-operation with subfraction 4 from the hexane-soluble fraction; *n* = 10). From the week after surgery, all mice were orally administered the fractions dissolved in 2% ethanol once a day for 12 weeks. Changes in body weight were measured weekly. All laboratory animals were treated according to the national regulations on the usage and welfare of animals and were approved by the Institutional Animal Care and Use Committee of Sungkyunkwan University (Suwon, Korea) prior to the experiments (approval no. SKKUIACUC2021-03-33-1).

### 2.8. Measurement of Bone Mineral Density (BMD), Lean Density, and Body Fat Density

The mean bone mineral density of the total body and right femur were detected once a month from OVX and at sacrifice using live dual-energy X-ray absorption spectrometry (DEXA) (Lunar PIXImus; GE, Fitchburg, MI, USA). Whole-body DEXA images derived from scanning were captured individually. The mean lean and fat densities of the body of each mouse were automatically calculated.

### 2.9. 9.4T MRI Scanning

9.4T MRI scanning for small animals was conducted with professional technical support from the Institute for Basic Science (IBS) Center for Neuroscience Imaging Research (IBS-R015-D1). The right femur, separated from each mouse, was immersed in Fluorinert FC-770 buffer (3M Electronics, USA) and sealed in 1.8 mL centrifuge tubes to maintain sample moisture without introducing a signal to the magnetic resonance image (MRI) [40]. 9.4T MRI was equipped with a Bruker console (AVANCE II, ParaVision software) and a horizontal bore, a Bruker gradient (BGA12S, 720 mT/m). The sample was secured in a birdcage radiofrequency coil and scout scanned coronally, transversely, and sagittally to cover the full size of each bone. Data collection was conducted in the sagittal plane, which provided an accurate measurement of the articular cartilage. To achieve high pixel resolution (50 mm) and high vibration stability in the sagittal plane, 12 scans of femur samples were continuously performed back-to-back using a two-dimensional (2D) gradient-echo fast-low-angle-shot sequence (5.4-millisecond echo time, 3000-millisecond repetition time, and 90 flip angle). This sequence was optimal for the visualization of articular cartilage in our settings.

### 2.10. Histology

The collected left femur from each mouse was fixed in 10% neutral buffered formalin (NBF) and then placed in a decalcifying solution (24.4% formic acid and 0.5 N sodium hydroxide) for 3 days. The bone tissues were subsequently embedded in paraffin. The sectioned (3–4 μm) tissues were stained with hematoxylin and eosin (H&E) or Safranin-O and observed under a light microscope (Nikon Corp., Minato City, Tokyo, Japan).

### 2.11. Biochemical Analysis

Biochemical analysis of the 14 indicators was conducted by a specialized analysis agency (DK Korea, Korea). Serum ALP, calcium (CA), and IP were identified as osteoporosis-related markers. T. cho., LDL, HDL, triglyceride (TG), glucose, and FFA were detected as related indicators. In addition, serum AST, ALT, BUN, creatine, and LDH were detected to diagnose liver and kidney disease and estimate recovery from physical fatigue.

### 2.12. Statistical Analysis

Each sample was tested in triplicate before the test, and all tests were repeated thrice. Data are presented as mean ± standard deviation (S.D.). Differences between the control and experimental groups were analyzed using a two-tailed unpaired Student’s *t*-test, and statistical significance was defined as *p* < 0.05.

## 3. Results

### 3.1. Effect of the H. rhamnoides Fruit Extract on Osteogenesis of Mesenchymal Stem Cell

**Figure 1:**
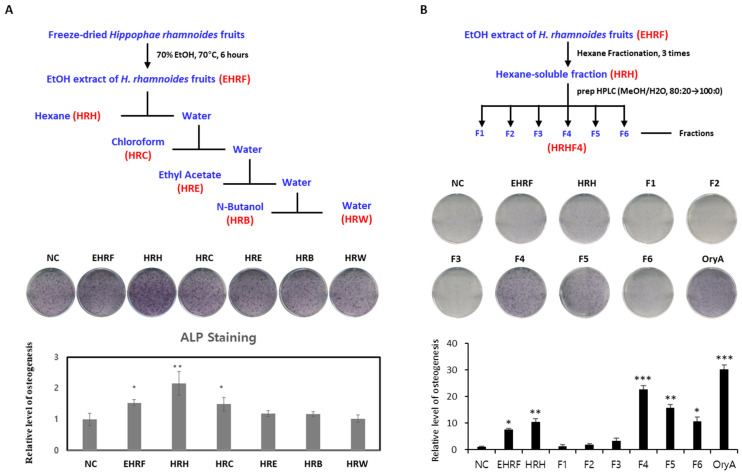
Evaluation of the effects of each fraction from the EtOH extract of *H. rhamnoides* fruits on osteogenesis. (**A**) Stimulatory effect of each solvent soluble fractions, derived from the process above, on the osteogenic differentiation of mesenchymal stem cells (MSCs) into osteoblasts. Fully differentiated C3H10T1/2 cells were stained with alkaline phosphatase (ALP) after 9 days of osteogenic differentiation with 50 μg/mL of each isolated extract. The relative levels were calculated by setting the negative control (NC) to 1. (**B**) Stimulatory effects of each extract, derived from the process above, on osteogenic differentiation of MSCs toward osteoblasts. Fully differentiated C3H10T1/2 cells were stained with ALP after 9 days of osteogenic differentiation with 50 μg/mL of each isolated extract. The relative levels were calculated by setting the negative control (NC) to 1. Oryzativol A (5 μM, 3.64 μg/mL), which has been proven to be effective in osteogenesis, was used as a positive control and marked as OryA. *, **, and *** indicate difference from nontreated control. * denotes 0.01 ≤ *p* ≤ 0.05; ** denotes 0.001 ≤ *p* < 0.01; and *** denotes *p* < 0.001.

Dried *H. rhamnoides* fruit powder was extracted using 70% ethanol at 70 °C and filtered. After evaporation of the filtrate, the EtOH extract of *H. rhamnoides* fruits (EHRF) was obtained. To determine the effect of EHRF on promoting osteogenesis, the effect of the extract on the differentiation of murine MSCs into osteoblasts was tested. C3H10T1/2, a mesenchymal stem cell line, was treated with 50 μg/mL of the extract during osteogenesis, and the differentiated cells were stained to demonstrate alkaline phosphatase (ALP) production, which is a representative marker of osteogenesis. EHRF significantly stimulated the differentiation of MSCs into osteoblast by 7.5 times (* *p* < 0.05) compared to that in the negative control (Figure 1A). In the next step, EHRF was resolved in distilled water and fractionated by a solvent partitioning process to obtain hexane-soluble (HRH), chloroform-soluble (HRC), ethyl acetate-soluble (HRE), and *n*-butanol-soluble (HRB) fractions, as well as water residue (HRW) (Figure 1A). C3H10T1/2 cells were treated with five fractions (HRH, HRC, HRE, HRB, and HRW) at a concentration of 50 μg/mL, and the degree of differentiation of these cells was measured. Cells treated with HRH showed a 10.4 times (** *p* < 0.01) increase in ALP staining compared to that in the negative control (Figure 1A). Our results showed that MSCs in cultures treated with HRH tended to differentiate effectively into osteoblasts. The active HRH was fractionated by preparative HPLC to obtain six subfractions (HRHF1–HRHF6). Among the six subfractions from HRH, HRHF4 showed the highest activity which was 22.6 times (*** *p* < 0.001) higher than that of the negative control, indicating an osteogenic effect similar to that of the positive control (oryzativol A, 5 μM), which showed 30.3 times (*** *p* < 0.001) higher activity compared to that of the negative control (Figure 1B).

**Figure 2:**
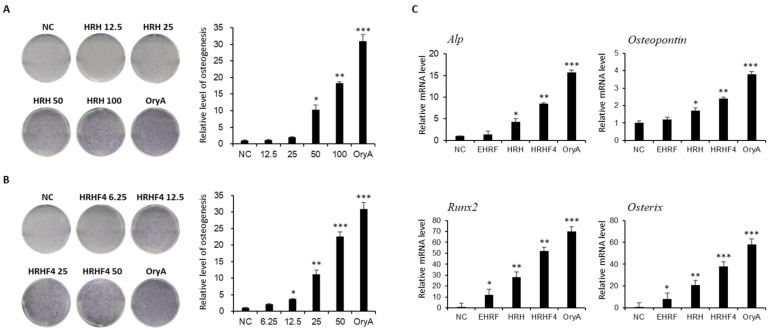
Evaluation of the effects of the hexane-soluble (HRH) and HRHF4 fractions on osteogenesis. (**A**) Concentration-dependent stimulatory effect of the hexane-soluble fraction from an ethanol extract of *H. rhamnoides* fruits (HRH) on osteogenic differentiation of C3H10T1/2 cells toward osteoblasts. Four different concentrations (12.5, 25, 50, and 100 μg/mL) of HRH were used for treatment to evaluate the concentration-dependent efficacy on osteogenic differentiation of C3H10T1/2 cells. The relative levels were calculated and compared using the same method as mentioned for Figure 1. (**B**) Concentration dependent stimulatory effect of Hexane-soluble fraction number 4 from ethanol extract of *H. rhamnoides* fruits (HRHF4) on osteogenic differentiation of C3H10T1/2 cells toward osteoblasts. Four different concentrations (6.25, 12.5, 25, 50 μg/mL) of HRHF4 were used to evaluate the concentration-dependent efficacy on osteogenic differentiation of C3H10T1/2 cells. The relative levels were calculated and compared using the same method as mentioned in Figure 1. (**C**) Relative mRNA expression of *Alkaline Phosphate (ALP)*, *Osteopontin*, *Runx2*, and *Osterix* in C3H10T1/2 osteoblasts incubated with 50 μg/mL of each extract or fraction (EHRF, HRH, HRHF4) during osteogenesis. * denotes *p* < 0.05, ** denotes *p* < 0.01, and *** denotes *p* < 0.001 vs. negative control, determined by the least significant difference test. Oryzativol A (5 μM, 3.64 μg/mL) was used as a positive control and marked as OryA.

Based on the results shown in Figure 1B, the differentiation experiment was repeated by treating HRH and HRHF4 at four different concentrations for concentration-dependent efficacy evaluation, and both types of HRH and HRHF4 showed efficacy closely related to increasing dose in osteoblast differentiation (Figure 2A,B). To determine whether the EtOH extract of *H. rhamnoides* fruits and their fractions affected the expression of the genes that induce osteogenesis, the relative mRNA expression levels of four representative genes (*Alp*, *Osteopontin*, *Runx2*, and *Osterix*) in C3H10T1/2 osteoblasts were analyzed by real-time PCR (Figure 2C). The PCR results confirmed that the extract of *H. rhamnoides* fruits and their fractions significantly upregulated the expression levels of the four genes related to osteogenesis. EHRF increased the expression of *Runx2* and *Osterix* by 12.5 times (* *p* < 0.05) and 8.5 times (* *p* < 0.05), respectively. HRH significantly upregulated the expression of all four genes; in particular, it increased the expression levels of *Runx2* and *Osterix* by 28.6 times (** *p* < 0.01) and 21.6 times (** *p* < 0.01), respectively. HRHF4 also significantly upregulated gene expression, increasing the expression of *Runx2* and *Osterix* by 52.5 times (** *p* < 0.01) and 38.7 times (*** *p* < 0.001), respectively. In addition, HRHF4 could promote the expression of *Alp* and *Osteopotin* by 8.4 times (** *p* < 0.01) and 2.4 times (** *p* < 0.01), respectively, showing that HRHF4 upregulated the expression of all four genes which are related to osteogenesis. These results demonstrate that HRHF4 is the active fraction from EHRF and that the active fraction promoted osteogenic gene expression during osteogenesis and significantly stimulated the differentiation of MSCs into osteoblasts.

### 3.2. Suppression of Body Weight Gain during Oral Administration of Active Fractions of H. rhamnoides in OVX Mice

**Figure 3:**
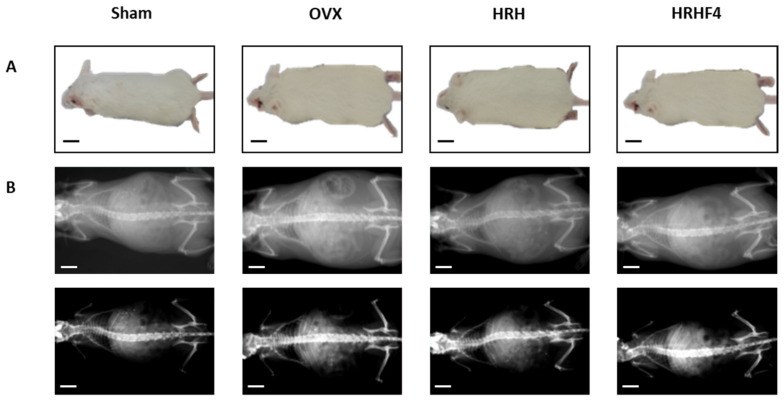
Representative images of each mouse group at the end of animal testing. (**A**) Whole body images of each mouse group. (**B**) whole body DEXA images of each mouse group. OVX, bilateral ovariectomy; HRH, Hexane-soluble fraction from ethanol extract of *H. rhamnoides* fruits; HRHF4, Hexane-soluble fraction number 4 from ethanol extract of *H. rhamnoides* fruits; DEXA, dual-energy X-ray absorptionmetry. Scale bar, 20 mm.

The weight of the mice that underwent ovariectomy continuously and dramatically increased after ovarian resection. Body weight was measured weekly for each mouse from the start until the end of oral administration of the active fractions (Table 1). The OVX group, during the 84 days of oral administration, gained 3.17 g, equivalent to 6.94% of the initial body weight, while the sham group without ovariectomy showed only a weight gain of 1.10 g, equivalent to 2.33% of the total body weight. During the same period, the increase in weight of the group of taking HRH 150 mg/kg and HRHF4 50 mg/kg was 2.31 g (* *p* < 0.05) and 1.70 g (** *p* < 0.01), respectively, which corresponded to 5.94% and 3.83% of their body weight. In particular, mice that ingested 50 mg/kg of HRHF4 showed a significant weight difference (* *p* < 0.05) compared to that of the OVX group at the final point. Considering the symptoms of weight gain in postmenopausal women along with the occurrence of osteoporosis, the results suggested that oral administration of the fractions from *H. rhamnoides* extract could alleviate the symptoms of menopause, including weight gain (Figure 3A).

### 3.3. Effect of the Active Fractions on Fat Density, Bone Mineral Density, and Lean Density in the OVX Mice

In addition to body weight, the bone mineral density, fat density, and lean density of osteoporosis-induced mice were measured at the start and end time points to evaluate the changes before and after administration of the active fractions (Table 1). Similar to body weight, compared to that in the sham group, the animals with ovarian resection showed significantly increased fat mass from the start of the study and showed a steady increase in fat density until the end of the study. The groups treated with HRH 150 mg/kg and HRHF4 50 mg/kg showed a decrease in fat density compared to that in the OVX group at the end of the study. In the 150 mg/kg group, the fat density decreased by 46.58% (* *p* < 0.05) before and after administration, and in the group taking HRHF4 50 mg/kg, the fat density decreased by 46.01% (* *p* < 0.05). Although a significant decrease in fat density was observed, the groups taking HRH 150 mg/kg and HRHF4 50 mg/kg demonstrated increased lean density during the administration period (HRH, 0.90%, * *p* < 0.05; HRHF4, 1.12%, ** *p* < 0.01). After administration of HRH and HRHF4, body weight increased slightly during 12 weeks. However, the increase was marginal compared to that of OVX control group. Fat density decreased while lean density increased, suggesting that the increase in lean was the cause of weight gain instead of fat. The above results indicate that the active fractions affected not only weight gain, but also fat and lean density during administration.

As a result of osteoporosis induction, OVX mice showed a significant decrease in bone mineral density (BMD) (** *p* < 0.01) (Table 1). Through BMD measurement using dual-energy X-ray absorptiometry (DEXA), images of the whole mouse body were obtained (Figure 3B). The image in the first row is taken to reveal the outline of the mouse body, indicating that the degree of obesity induced by OVX surgery was improved after administration of an active fraction. The image in the second row was shown by adjusting the brightness and contrast so that the spine and leg bones were more prominent than the shape of the body. Although it is difficult to visually compare the difference in bone mineral density through the image, the BMD level is quantified based on the image and shown in Table 1 and Table S1. During the study, the average BMD of the OVX group decreased by 0.0009 g/cm^2^, while that of the HRH intake group increased by 0.0033 g/cm^2^ (* *p* < 0.05) and that of the HRHF4 intake group increased by 0.0059 g/cm^2^ (** *p* < 0.01). In addition, femur BMD was measured by separating the right femur of mice in each group (Table S1). The BMD of the right femur in the HRH intake group was 0.0882 g/cm^2^ (* *p* < 0.05) and that of the HRHF4 intake group was 0.0905 g/cm^2^ (** *p* < 0.01), indicating a trend similar to that of the whole-body BMD. The right femur of each mouse was weighed before and after drying in an oven to remove any moisture from the tissue. In both wet and dry conditions, the HRH and HRHF4 intake groups showed significant differences (* *p* < 0.05) compared to the OVX group (Table S1). The weight of the femur relative to total body weight was 0.226% (*p < 0.05) in the HRH intake group and 0.237% (** *p* < 0.01) in the HRHF4 intake group. These results confirmed that the active fractions significantly recovered BMD and bone weight in mice with osteoporosis induced by ovarian resection.

### 3.4. Suppressive Effect of the Active Fractions on Deposition of Bone Marrow Fat through 9.4T Magnetic Resonance Imaging (MRI) Analysis

**Figure 4:**
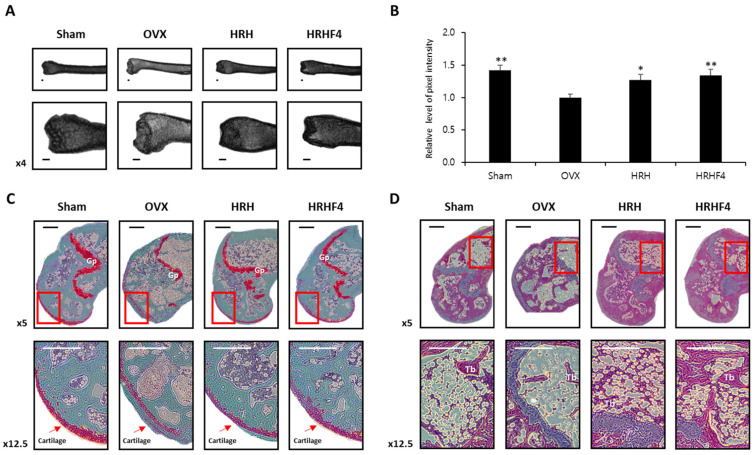
Magnetic-resonance-imaging (MRI) and histological images of the left femur from each mouse group. (**A**) 9.4T MRI. Image data of the left femur of mice from each group. (**B**) Relative level of pixel intensity of the femur captured with MRI images. The pixel intensity shown through MRI results was quantified using the Image J (Java) program. * denoted *p* < 0.05, ** denotes *p* < 0.01 vs. OVX control, determined by the least significant difference test. (**C**) Representative histological profiles of the left femur, captured from each mouse group after Safranin O staining. To observe the cartilage of the femur in detail, it was enlarged and captured step by step. (**D**) Representative histological images of the left femur captured from each mouse group after hematoxylin and eosin staining. To observe the femur bone marrow in detail, it was enlarged and captured step by step. OVX, bilateral ovariectomy; HRH, Hexane-soluble fraction from ethanol extract of *H. rhamnoides* fruits; HRHF4, Hexane-soluble fraction number 4 from ethanol extract of *H. rhamnoides* fruits; Tb, trabecular bone; Gp, growth plate. Scale bar, 250 μm.

DEXA has been a clinical baseline standard for diagnosing osteoporosis and evaluating fracture risks for decades. Recently, high-resolution MRI has provided data to supplement DEXA-based analysis [39]. Investigation of fat properties through MRI scans can allow early diagnosis of osteoporosis. The more bone marrow fat there is, the brighter it appears, and it is possible to quantify and compare it between individuals [41]. Images of the right femur of each mouse group taken with 9.4T MRI are shown in Figure 4. The pixel intensity of each femur image was quantified using Image J (Java) software to show the relative value (Figure 4B). As described above, 9.4T MRI confirmed that the active fractions, HRH and HRHF4, reduced bone marrow fat in the mouse femur during oral administration and enhanced the pixel intensity of the femur MRI image by 27.20% and 34.60%, respectively. These results confirmed that the active fractions were effective in reducing lipogenesis and increasing bone density.

### 3.5. Protective Effects of the Active Fractions on the Breakdown of the Bone Structure in the Osteoporosis-Induced Femur

To analyze the breakdown of bone structure due to osteoporosis progression, the left femur of each mouse was isolated and stained with safranin O (Figure 4C) or hematoxylin and eosin (Figure 4D). In contrast to the sham control, cartilage on the femur of OVX animals was damaged and seldom remained. Moreover, the hematopoietic tissue was significantly decreased in the OVX group compared with that observed in the sham group. In the OVX groups, the trabecular bone was thin and the network structure was severely disrupted with expanded spaces, in contrast to the trabecular bone in the sham group, which exhibited a regularly arranged pattern with a thick and dense network. In addition, the number of trabeculae was significantly reduced in the OVX animals. However, in the HRH and HRHF4 ingestion groups, uptake of active fractions improved cartilage damage and suppressed disruption of the trabecular bone structure. The results of the histological profiles showed that the active fractions protected the trabecular cartilage and bone structure in osteoporosis-induced animals.

### 3.6. Effect of the Active Fractions on Expression of Osteogenic MRNAs and Proteins in the OVX Mice

**Figure 5:**
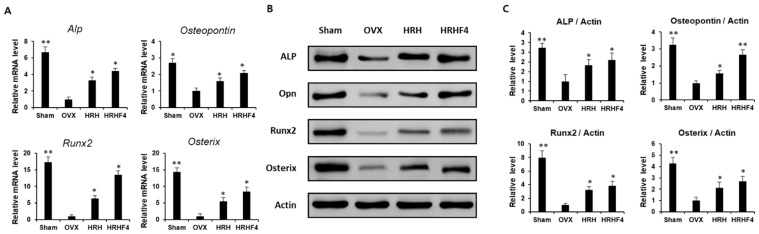
Expression of osteogenic mRNAs and proteins in mouse tibia samples. (**A**) Relative osteogenic mRNA expression of *Alkaline Phosphate (ALP)*, *Osteopontin (OPN)*, *Runx2*, and *Osterix* in each sample, obtained by pulverizing the both tibia of each mouse after animal sacrifice. (**B**) Protein expression of osteogenic genes including *ALP*, *OPN*, *Runx2*, and *Osterix,* and *β-actin*, a housekeeping gene in each sample, were obtained by pulverizing both the tibiae of each mouse after animal sacrifice. (**C**) The expression shown through Western blot results was quantified through the ImageJ (Java) software. * denotes *p* < 0.05, ** denotes *p* < 0.01 vs. OVX control, determined by the least significant difference test compared to level of *β-actin*, a housekeeping protein expression. OVX, bilateral ovariectomy; HRH, Hexane-soluble fraction from ethanol extract of *H. rhamnoides* fruits; HRHF4, Hexane-soluble fraction number 4 from ethanol extract of *H. rhamnoides* fruits.

Both tibiae from each mouse were separated and pulverized after sacrifice. mRNA and protein were extracted from the separated tissues to analyze the gene expression related to osteogenesis. The amount of mRNA expression was analyzed by real-time PCR (Figure 5A), and the protein expression was analyzed by Western blotting (Figure 5B). The band of each protein obtained through Western blotting was quantified using Image J (Java) software (Figure 5C). The expression of representative osteogenic biomarkers, including *ALP*, *Osteopontin*, *Runx2*, and *Osterix*, was significantly reduced in the tibia obtained from mice in the OVX group. However, the HRH and HRHF4 treated groups significantly recovered (* *p* < 0.05) the expression of the four types of osteogenic mRNAs. The groups administered the active fraction also significantly recovered the expression of the four representative types of osteogenic proteins. In the HRH group, the mRNA expression of *ALP*—a hallmark of the osteoblast phenotype—was increased 3.3-fold compared to that in the OVX-control group, and the expression of ALP protein was increased 1.8-fold. In the HRHF4-uptake group, the expression of *ALP* mRNA increased 4.4-fold compared to that in the OVX-control group, and the expression of ALP protein increased 2.1-fold. The mRNA expression of *Runx2*, an osteogenic commitment marker, increased by 6.4 times and 13.5 times that in the HRH and HRHP4-uptake groups, respectively. The protein expression of Osteopontin increased by 1.6 times and 2.7 times in the HRH and HRHF4-uptake groups, respectively (** *p* < 0.01). In particular, HRHF4 increased *Osterix* gene expression and protein expression by 8.5-fold and 2.7-fold, respectively. Based on the above results, the expression levels of osteogenic mRNAs and proteins in the mouse tibia were significantly recovered through the ingestion of the active fractions after osteoporosis was induced. This can be interpreted as the active fractions activating osteogenic gene expression in osteoporosis-induced bone, resulting in active bone metabolism, as in normal animals.

### 3.7. Changes in Serum Biochemistry by the Active Fractions

**Figure 6:**
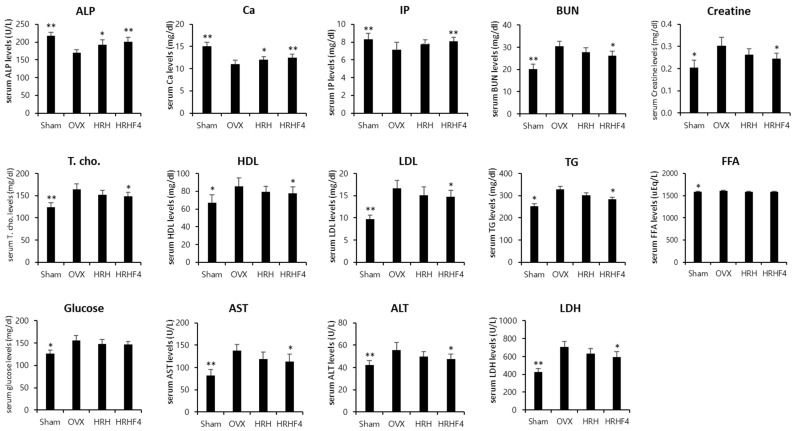
Serum biochemistry analysis of each mouse group. Values are expressed mean ± standard deviation (*n* = 10). Two different types and doses of *H. rhamnoides* extracts were orally administered once a day for the next 84 days from the time of 84 days of osteoporosis induction after OVX surgery. * denotes *p* < 0.05, ** denotes *p* < 0.01 vs. OVX control, determined by the least significant difference test. OVX, bilateral ovariectomy; HRH, Hexane-soluble fraction from ethanol extract of *H. rhamnoides* fruits; HRHF4, Hexane-soluble fraction number 4 from ethanol extract of *H. rhamnoides* fruits; ALP, alkaline phosphatase; Ca, calcium; IP, inorganic phosphorus; BUN, blood urea nitrogen; HDL, high density lipoprotein cholesterol; LDL, low density lipoprotein cholesterol; TG, triglyceride; FFA, free fatty acid; AST, Aspartate Aminotransferase; ALT, alanine aminotransferase; LDH, lactate dehydrogenase.

Significant reductions in serum ALP, calcium (Ca), and phosphate (IP) levels and significant increases in serum total cholesterol (T. cho), high density lipoprotein cholesterol (HDL), low density lipoprotein cholesterol (LDL), triglyceride (TG), free fatty acid (FFA), and glucose levels were observed in the OVX control group compared with those in the sham control group, indicating that osteoporosis was induced normally through ovarian resection surgery (Figure 6). Significant increases in serum blood urea nitrogen (BUN), creatinine, aminotransferase (AST), alanine aminotransferase (ALT), and lactate dehydrogenase (LDH) were observed in the OVX control group compared with that in the sham control group, indicating that diseases of the liver and kidneys occurred through the induction of osteoporosis. However, HRH and HRHF4 intake groups showed significant effects on the recovery of the serum ALP levels, which decreased by 27.9% (** *p* < 0.01) due to osteoporosis induction, by 13.2% (* *p* < 0.05) and 18.1% (** *p* < 0.01), respectively. Serum calcium levels decreased by as much as 36.2% (** *p* < 0.01) and recovered by 8.5% (* *p* < 0.05) and 13.1% (** *p* < 0.01) in the HRH and HRHF4 intake groups, respectively. The increased level of serum calcium according to HRH and HRHF4 intake was within the normal range of serum calcium in rodents. The IP level, which was 16.4% (** *p* < 0.01) after ovarian resection, also significantly recovered by 8.8% (** *p* < 0.01) and 13.4% (** *p* < 0.01) after ingestion of HRH and HRHF4, respectively. In addition, it was identified that various serum cholesterol and lipid-related levels that rapidly increased after 12 weeks of osteoporosis induction were significantly lower in the active fraction intake group. In particular, in the HRHF4 intake group, serum T. cho, TG, HDL, and LDL levels were significantly reduced by 9.6% (* *p* < 0.05), 13.7% (* *p* < 0.05), 9.1% (* *p* < 0.05), and 11.5% (* *p* < 0.05), respectively. When the active fractions were ingested, the levels of serum BUN, creatine, AST, ALT, and LDH were also significantly reduced, showing a possibility of the treatment of diseases in organs caused by osteoporosis. Based on the above serum biochemical analysis results, the administration of active fractions increased the serum levels of bone health-related biomarkers, which were decreased by osteoporosis, and reduced the serum levels of obesity and inflammation-related biomarkers, which were increased by osteoporosis.

## 4. Discussion

*H. rhamnoides*, known as sea buckthorn, has been shown to have various physiological activities, including anti-inflammatory and antioxidant properties, and has been used to ameliorate several diseases and improve various pathological symptoms, including obesity [37]. It has been reported that the leaves and seeds of sea buckthorn have anti-obesity effects in an in vitro model of adipocyte differentiation and in an in vivo model of high-fat diet-induced obesity [42,43,44]. Freeze-dried powder of sea buckthorn fruit prevented obesity and abnormal lipid metabolism by modulating the gut microbiota of mice fed a high-fat diet [45]. Concurrently, controversial results have been reported that oil from sea buckthorn fruit promotes the proliferation of preadipocytes and differentiation into adipocytes [46]. In contrast, no studies have shown that *H. rhamnoides* fruit suppressed menopause-induced lipid accumulation and body fat increase. Above all, no studies have investigated the efficacy of *H. rhamnoides* fruit on osteoporosis, a representative symptom of menopause.

We have shown for the first time that some substances isolated from the *H. rhamnoides* fruit promote osteocyte differentiation [38]. In previous studies, we confirmed that compounds isolated from the hydrophilic part of *H. rhamnoides* ethanol extract had a marginal effect on osteogenesis in the MSC line, C3H10T1/2. MSCs in the bone marrow differentiate into adipocytes and osteocytes. Therefore, in this study, we tested whether the hydrophobic part of the *H. rhamnoides* extract can stimulate osteogenic differentiation of MSCs. Bone metabolism is always in constant dynamic equilibrium, and it maintains homeostasis with a physiological balance between bone formation and bone resorption through interactions of osteoblasts, osteocytes, and osteoclasts [47]. However, when bone metabolism imbalance occurs, bone absorption is greater than bone formation, reducing bone density and causing osteoporosis [48]. The majority of the drugs used for prevention and treatment of osteoporosis decrease the bone resorption-antiresorptive agents such as hormone replacement therapy, selective estrogen receptor modulators (SERM); Raloxifene, bisphosphonates; Alendronate, Risedronate, Ibandronate, and Zoledronic acid, human monoclonal antibody against RANKL; Denosumab [49]. Through in vitro testing, the effect on the promotion of osteoblast differentiation for C3H10T1/2 cells was investigated. Micro-environmental changes lead to differences in gene expression in MSC differentiation, where alterations in the expression of related genes may interfere with the balance between osteoblast progenitor and adipocyte progenitor cells in osteoporosis patients [50,51]. Therefore, we focused on the fact that bone metabolism can be balanced by promoting bone formation in the skewed equilibrium state of osteoporosis.

Postmenopausal women have an increased risk of osteoporosis due to excessive osteoclast activity followed by estrogen deficiency [52,53]. Therefore, we conducted this study to determine whether *H. rhamnoides* berries effectively stimulate osteogenesis and maintain the equilibrium of bone metabolism prevent bone loss in OVX mice with osteoporosis, a model for human menopause. Resection of the ovaries in rodent animals causes weight gain owing to excessive fat accumulation caused by estrogen deficiency [54]. After ovariectomy, the levels of lipoprotein lipase, the enzyme that hydrolyzes triglycerides in plasma lipoproteins and releases fatty acid nutrients to vital tissues, increase, resulting in fat accumulation in adipocytes [55,56]. The whole-body weight of the mice in the postmenopausal osteoporosis model induced by ovarian resection was higher than that of normal mice. In addition, osteoporotic animals showed an increase in body fat density compared to that of normal mice, implying that fat accumulation, one of the postmenopausal symptoms, was reproduced in mice that underwent ovariectomy. In contrast, the weight of the femur in the postmenopausal osteoporosis model was lower than that in the normal mouse femur. We found that the weight of the femur in the OVX group was significantly reduced compared to that in the sham group, and the weight of the femur in the group treated with *H. rhamnoides* fruit extract was significantly higher than that in the OVX group. These results indicate that the administration of *H. rhamnoides* fruit extract suppresses weight loss in the femur, suggesting that *H. rhamnoides* promotes bone formation in an OVX-induced osteoporosis model.

BMD is defined as the average concentration of minerals per unit area, and it is related to the minimum force required to induce a fracture. Thus, it serves as an index for predicting the risk of fracture and is used as the gold standard for osteoporosis [57]. In this study, changes in BMD of the whole bone and femur were significantly decreased in the OVX group compared to that in the Sham group and significantly increased in the test group treated with *H. rhamnoides* fruit extract compared to that in the OVX group (Table 1). Bone homeostasis is maintained by a balance between bone resorption in osteoclasts and bone formation in osteoblasts [58]. According to previous studies, osteogenic signals in osteoblasts induce programmed cell death in osteoclasts [59]. In osteoblasts derived from an ovariectomized osteoporosis rat model, the expression of this signal is reduced, resulting in a reduction in the programmed apoptosis of osteoclasts and an increase in bone reabsorption [60]. Disparity in the interaction between osteoclasts and osteoblasts induces bone diseases such as osteoporosis by increasing the rate of bone resorption rather than bone formation.

ALP expression is one of the major molecular events in the early stages of osteoblast differentiation and is related to the promotion of bone mineralization and regulation of osteoclasts [60,61]. A significant amount of ALP mRNA has also been reported to be expressed in normal human osteoblasts under controlled culture conditions [62]. ALP is a potential Ca^2+^ transporter that is thought to play an important role in bone formation by hydrolyzing mineralization inhibitors [63]. During the differentiation of MSCs into osteocytes, ALP mRNA expression was significantly increased in cells treated with the *H. rhamnoides* fruit extract compared to that in untreated control cells (Figure 1 and Figure 6 and Table 1). Additionally, Alp mRNA and protein levels were significantly decreased in the tissues of OVX animals compared to those in normal tissues; however, the expression of alp mRNA and protein was significantly increased in the tissues of animals that were treated with *H. rhamnoides* fruit extract (Figure 5).

Runt-related transcription factor-2 (RunX2), a Runt DNA-binding domain-containing protein, plays an important role in the differentiation of MSCs into osteoblasts in humans and mice [64]. The expression of RunX2 is strongly related to osteoporosis due to menopause, and it has been reported that the expression of *RunX2* is significantly reduced in patients with osteoporosis [65]. The expression of osteogenic differentiation genes, including *RunX2*, is also closely related to bone mineral density, and it has been reported that the expression of *RunX2* increases or decreases in proportion to physiological and pathological changes in mineral density [66,67]. We also observed decreased expression of osteoblastic genes (Figure 5), such as *Runx2* and *Osterix*, and a reduction in BMD (Table 1) in OVX mice. The expression of osteogenesis-promoting genes was restored in the tissues of animals whose BMD was increased by ingestion of the *H. rhamnoides* fruit extract, suggesting that there is a close relationship between osteogenic gene expression and BMD. Activation of Runx2 in MSCs induces the expression of a master regulator of osteoblast differentiation, *Osterix*, which in turn induces the expression of downstream osteogenic genes encoding glycoproteins, such as collagen type I (COL1A1), bone alkaline phosphatase (bALP), and osteopontin (OPN) [67]. As the expression of *Runx2* in animal tissues, which decreased with osteoporosis induction, was increased after ingestion of *H. rhamnoides* fruit extract, it was observed that the mRNA and protein expression of osterix, alp, and OPN were also increased (Figure 5).

The imbalance between bone resorption and mineral accumulation is a classic pathological phenomenon of osteoporosis [68], and to differentiate and maintain bone tissue, a balance between bone resorption and bone deposition is required [69]. Bone formation is accompanied by calcium accumulation followed by bone mineralization [70] and we found that ingestion of the *H. rhamnoides* extract increased the accumulation of minerals, including calcium, in the bone tissues of OVX mice.

## 5. Conclusions

In conclusion, the fruit extract of *H. rhamnoides* improved bone mineral accumulation by promoting the expression of osteogenic genes, such as *Runx2* and *Osterix*, and stimulating osteocyte differentiation. In this study, we confirmed that *H. rhamnoides* extract can be considered a possible therapeutic agent for the treatment of postmenopausal osteoporosis by activating bone metabolism and preventing the breakdown of bone tissue. Further study is required to identify the relevant active compounds from the active fraction.

## Figures and Tables

**Table 1:** Whole body weight, bone mineral density, body fat, and lean density changes of each mouse group.

Variable	Whole Body Weight (g)			Bone Mineral Density (g/cm^2^)			Fat Density (% of Body Mass)			Lean Density (% of Body Mass)		
Groups	Initial	Final	Changes	Initial	Final	Changes	Initial	Final	Changes	Initial	Final	Changes
Controls												
Sham	47.12 ± 4.72	48.22 ± 4.65	1.1 ± 0.11 **	0.0969 ± 0.0019 **	0.0962 ± 0.0018 ***	−0.0007 ± 0.001	40.42 ± 3.01 **	40.65 ± 3.56 **	0.23 ± 0.97	59.58 ± 3.01 **	59.35 ± 3.56 **	−0.23 ± 0.97
OVX	45.68 ± 4.37	48.85 ± 4.39	3.17 ± 0.43	0.0836 ± 0.005	0.0827 ± 0.005	−0.0009 ± 0.0003	46.91 ± 1.26	47.91 ± 1.75	1.00 ± 0.79	53.09 ± 1.26	52.09 ± 1.75	−1.00 ± 0.79
Active fractions from *H. rhamnoides* extract												
HRH	45.85 ± 7.47	48.16 ± 7.44	2.31 ± 0.66 *	0.0837 ± 0.0036	0.0870 ± 0.004 *	0.0033 ± 0.0019 *	47.48 ± 1.20	46.58 ± 1.28 *	−0.90 ± 0.47 *	52.52 ± 1.20	53.42 ± 1.28 *	0.90 ± 0.47 *
HRHF4	44.37 ± 6.61	46.07 ± 6.17 *	1.7 ± 0.47 **	0.0833 ± 0.0040	0.0892 ± 0.0036 **	0.0059 ± 0.0009 **	47.13 ± 1.92	46.01 ± 1.69 *	−1.12 ± 0.95 **	52.87 ± 1.92	53.99 ± 1.69 *	1.12 ± 0.95 **

Values are expressed as mean ± standard deviation (*n* = 10). Two different types and doses of *H. rhamnoides* extracts were orally administered, once a day for 84 days from 12 weeks after OVX surgery. * *p* < 0.05, ** *p* < 0.01, *** *p* < 0.001 vs. OVX control, determined by the least significant difference test. OVX, bilateral ovariectomy; HRH, Hexane-soluble fraction from ethanol extract of *H. rhamnoides* fruits (150 mg/kg); HRHF4, Hexane-soluble fraction number 4 from ethanol extract of *H. rhamnoides* fruits (50 mg/kg).

## Data Availability

Not applicable.

## Supplementary Materials

The following supporting information can be downloaded at: https://www.mdpi.com/article/10.3390/nu14173604/s1, Table S1: Bone mineral density and weight of the right femur of each mouse group.
