# Supplementary material for: Anti-Osteoporosis Effects of the Fruit of Sea Buckthorn (Hippophae rhamnoides) through Promotion of Osteogenic Differentiation in Ovariectomized Mice
Source: Nutrients. 2022 Aug 31;14(17):3604. doi: 10.3390/nu14173604 (PMC9460184; doi:10.3390/nu14173604)
Supplement: Supplementary file 1 [file nutrients-14-03604-s001.zip › nutrients-1831567-supplementary.pdf]

nutrients

MDPI

Supplementary Materials

Anti-Osteoporosis Effects of the Fruit of Sea Buckthorn (*Hippophae rhamnoides*) through Promotion of Osteogenic Differentiation in Ovariectomized Mice

Kun Hee Park [1,†], Joo-Hyun Hong [2,†], Seon-Hee Kim [3], Jin-Chul Kim [4], Ki Hyun Kim [2,*] and Ki-Moon Park [1,*]

1 Department of Food Science and Biotechnology, Sungkyunkwan University, Suwon 16419, Korea
2 School of Pharmacy, Sungkyunkwan University, Suwon 16419, Korea
3 Sungkyun Biotech Co., Ltd., Suwon 16419, Korea
4 KIST Gangneung Institute of Natural Products, Natural Product Informatics Research Center, Gangneung 25451, Korea
* Correspondence: khkim83@skku.edu (K.H.K.); pkm1001@skku.edu (K.-M.P.); Tel.: +82-31-290-7700 (K.H.K.); +82-31-290-7806 (K.-M.P.)
† These authors contributed equally to this study.

Table S1. Bone mineral density and weight of the right femur of each mouse group.

Variable	Bone Mineral Density (g/m^2)	Absolute Bone Weight (g)		Relative Bone Weight (% of Body Weight)	
Groups		Wet	Dry	Wet	Dry
Controls					
Sham	0.0962±0.0038**	0.157±0.0008**	0.116±0.0004**	0.326±0.0021**	0.240±0.0015**
OVX	0.0832±0.0057	0.133±0.0007	0.099±0.0003	0.272±0.0023	0.202±0.0014
Active fractions from *H. rhamnoidesex-tracts*					
HRH 150mg/kg	0.0882±0.0064*	0.142±0.0013*	0.106±0.0009*	0.303±0.0024*	0.226±0.0016*
HRHF4 50mg/kg	0.0905±0.0054**	0.147±0.0007*	0.109±0.0004*	0.319±0.0019**	0.237±0.0012**

Values are expressed mean ± standard deviation (n=10). Two different types and dosages of H. rhamnoides extracts were orally administered, once a day for 84 days from 12 weeks after OVX surgery. $*p < 0.05$, $**p < 0.01$ vs. OVX control, determined by least significant difference test. OVX, bilateral ovariectomy; HRH, Hexane-soluble fraction fromethanol extract of H. rhamnoides fruits; HRHF4, Hexane-soluble fraction number 4 from ethanol extract of H. rhamnoides fruits.
